# *Lactiplantibacillus plantarum* LOC1 Isolated from Fresh Tea Leaves Modulates Macrophage Response to TLR4 Activation

**DOI:** 10.3390/foods11203257

**Published:** 2022-10-18

**Authors:** Masahiko Suzuki, Leonardo Albarracin, Yuji Tsujikawa, Kohtaro Fukuyama, Iwao Sakane, Julio Villena, Haruki Kitazawa

**Affiliations:** 1 Central Research Institute, *ITO EN* Ltd., Shizuoka 421-0516, Japan; 2 Laboratory of Animal Food Function, Graduate School of Agricultural Science, Tohoku University, Sendai 980-8576, Japan; 3 Laboratory of Immunobiotechnology, Reference Centre for Lactobacilli (CERELA-CONICET), Tucuman CP4000, Argentina; 4 International Education and Research Center for Food and Agricultural Immunology (CFAI), Graduate School of Agricultural Science, Tohoku University, Sendai 980-8576, Japan

**Keywords:** *Lactiplantibacillus plantarum*, LOC1, tea leaves, immunobiotic, macrophages, TLR4, genomics

## Abstract

Previously, we demonstrated that *Lactiplantibacillus plantarum* LOC1, originally isolated from fresh tea leaves, was able to improve epithelial barrier integrity in in vitro models, suggesting that this strain is an interesting probiotic candidate. In this work, we aimed to continue characterizing the potential probiotic properties of the LOC1 strain, focusing on its immunomodulatory properties in the context of innate immunity triggered by Toll-like receptor 4 (TLR4) activation. These studies were complemented by comparative and functional genomics analysis to characterize the bacterial genes involved in the immunomodulatory capacity. We carried out a transcriptomic study to evaluate the effect of *L. plantarum* LOC1 on the response of murine macrophages (RAW264.7 cells) to the activation of TLR4. We demonstrated that *L. plantarum* LOC1 exerts a modulatory effect on lipopolysaccharide (LPS)-induced inflammation, resulting in a differential regulation of immune factor expression in macrophages. The LOC1 strain markedly reduced the LPS-induced expression of some inflammatory cytokines (*IL-1β*, *IL-12*, and *CSF2*) and chemokines (*CCL17*, *CCL28*, *CXCL3*, *CXCL13*, *CXCL1*, and *CX3CL1*), while it significantly increased the expression of other cytokines (*TNF-α*, *IL-6*, *IL-18*, *IFN-β*, *IFN-γ*, and *CSF3*), chemokines (*IL-15* and *CXCL9*), and activation markers (*H2-k1*, *H2-M3*, *CD80*, and *CD86*) in RAW macrophages. Our results show that *L. plantarum* LOC1 would enhance the intrinsic functions of macrophages, promoting their protective effects mediated by the stimulation of the Th1 response without affecting the regulatory mechanisms that help control inflammation. In addition, we sequenced the LOC1 genome and performed a genomic characterization. Genomic comparative analysis with the well-known immunomodulatory strains WCSF1 and CRL1506 demonstrated that *L. plantarum* LOC1 possess a set of adhesion factors and genes involved in the biosynthesis of teichoic acids and lipoproteins that could be involved in its immunomodulatory capacity. The results of this work can contribute to the development of immune-related functional foods containing *L. plantarum* LOC1.

## 1. Introduction

*Lactiplantibacillus plantarum* are nomadic lactic acid bacteria (LAB) [1] and are known for their significant intraspecific versatility. This species of LAB is frequently isolated from dairy foods as well as fresh and fermented plant and meat. *L. plantarum* are also inhabitants of the gastrointestinal and urogenital tracts of humans and animals [2]. Consistent with its environmental range, *L. plantarum* strains have larger genomes compared with other species of LAB [3]. *L. plantarum* is widely used in the industry to produce numerous fermented foods, and certain strains are used as probiotics for the improvement of human and animal health.

Some *L. plantarum* strains have been shown to exert immunomodulatory effects on the host. Studies have demonstrated that strains such as *L. plantarum* CRL1506, MPL16 and LRCC5310 are able to regulate the Toll-like receptor 3 (TLR3)-mediated immune response in the intestinal mucosa and to increase the production of interferon (IFN)-γ and IFN-β as well as antiviral factors increasing the protection against viral infections [4,5,6,7]. *L. plantarum* YU was shown to strongly augment IL-12 production by intestinal antigen-presenting cells [8], which was associated with TLR2 stimulation. On the other hand, it was reported that *L. plantarum* strains are also able to regulate TLR4-mediated immunity. It was shown that the exopolysaccharide (EPS) of *L. plantarum* L-14 is able to regulate the inflammatory response triggered by lipopolysaccharide (LPS) administration to mouse RAW 264.7 macrophages [9]. The EPS from the L-14 strain reduced the expression of the inducible nitric oxide synthase and diminished the proinflammatory mediators interleukin (IL)-6, IL-1β and tumor necrosis factor (TNF)-α. *L. plantarum* L15 was shown to reduce the expression of TLR4, MyD88 and genes related to the NF-κB signaling pathway, inducing increased protection against ulcerative colitis [10], while mice treated with a mixture of three *L. plantarum* strains (KLDS 1.0318, KLDS 1.0344, and KLDS 1.0386) had significantly lower production of intestinal TNF-α, IL-6 and IL-12 and reduced gut tissue injury after the challenge with LPS [11]. Thus, the immunomodulatory probiotic or immunobiotic *L. plantarum* strains, particularly those with the ability to regulate TLR-mediated immune responses, are interesting alternatives to develop functional foods with the capacity to improve immune health [4,5,12].

Members of the *Lactobacillus* group have been isolated from fermented tea leaves including the species *Lactiplantibacillus pentosus* [13], *Limosilactobacillus fermentum* [14] and *L. plantarum* [15]. Meanwhile, the isolation of *Lactobacillus* strains from fresh tea leaves (*Camellia sinensis*, Theaceae) has been less explored [16]. In this regard, we recently isolated LAB strains from fresh tea leaves and evaluated the impact of selected strains on the intestinal barrier integrity using co-culture in vitro models of the small and large intestine [17]. Among the studied strains, *L. plantarum* LOC1 was able to improve epithelial barrier integrity and the expression of occludin and mucin genes in Caco-2/HT29-MTX mono- or co-cultures challenged with dextran sodium sulfate (DSS). These results showed that *L. plantarum* LOC1 is an interesting probiotic candidate with beneficial effects on the intestinal barrier.

To the best of our knowledge, few studies have evaluated the potential probiotic and immunobiotic properties of *L. plantarum* strains from fresh tea leaves. Carrying out these studies would be of great importance to find microorganisms with beneficial properties for health, which also have biotechnological properties not found in bacteria of dairy origin. Therefore, the objective of this work was to continue characterizing the potential probiotic properties of the LOC1 strain, focusing on its immunomodulatory properties in the context of innate immunity triggered by TLR4 activation. These studies were complemented by comparative and functional genomics analysis to characterize the bacterial genes involved in the immunomodulatory capacity.

## 2. Materials and Methods

### 2.1. Microorganisms

*Lactiplantibacillus plantarum* strains LOC1 and LOC3 were isolated from fresh tea leaves from a Kagoshima tea plantation in Japan [17]. *Lacticaseibacillus rhamnosus* CRL1505 and *L. plantarum* CRL1506 belong to CERELA-CONICET Culture Collection (Tucuman, Argentina) and were originally isolated from goat milk [6]. Stock cultures were stored at −80 °C in De Man, Rogosa and Sharpe (MRS) broth (Oxoid, Basingstoke, UK) broth containing 30% glycerol. Lactobacilli were propagated twice in MRS broth prior to use. Bacteria were grown overnight anaerobically at 37 °C in MRS broth. Lactobacilli from the stationary phase were harvested by centrifugation at 8000× *g*, followed by two washes with PBS.

### 2.2. Cell Cultures

RAW264.7, a mouse macrophage cell line (RIKEN Cell Bank, Tsukuba, Japan), was cultured in high-glucose Dulbecco’s modified Eagle medium (DMEM) (Thermo Fisher Scientific, Tokyo, Japan) containing 10% fetal bovine serum (FBS), 100 U/mL penicillin, and 100 μg/mL streptomycin (Thermo Fisher Scientific, Tokyo, Japan) at 37 °C in a humidified incubator containing 5% CO_2_.

Mouse intestinal epithelial (MIE) cells were maintained in Dulbecco’s modified Eagle’s medium (DMEM) (Thermo Fisher Scientific, Tokyo, Japan) supplemented with 10% fetal calf serum, 100 U/mL streptomycin, and 100 mg/mL penicillin at 37 °C in an atmosphere of 5% CO_2_. MIE cells grow rapidly and have a cobblestone morphology, which is a typical feature of intestinal epithelial cells [18].

### 2.3. Cell Stimulation with Lactobacilli and LPS

RAW264.7 cells were plated at a density of 5 × 10^5^ cells/well in 12-well plates and cultured for overnight. After changing medium, macrophages were cultivated for 3 h in the absence or presence of lactobacilli (5 × 10^7^ cells/mL). For LPS challenge experiments, RAW264.7 cells were cultivated overnight as described above, and after changing medium, lactobacilli (1 × 10^8^ cells/mL) were added to stimulate the cells for 24 h. Then, wells were washed with medium 3 times to eliminate lactobacilli and stimulated with LPS (50 ng/mL) for 3 h.

For the MIE–macrophage co-culture system, the Transwell culture system was used. MIE cells were seeded in the apical surface at a concentration of 2.5 × 10^5^ cells/well in 12-well tissue culture plates (Corning, pore size 0.4 µm) and cultured for three days. RAW264.7 cells were plated at a density of 5 × 10^5^ cells/well in 12-well plates and cultured overnight. After incubation, both cells were overlaid. For the evaluation of the immunomodulatory activity of lactobacilli in the MIE–immune cell co-culture system, the apical surface containing MIE cells was stimulated with lactobacilli strains (1 × 10^8^ cells/mL) for 48 h. Then, the basolateral surface containing RAW264.7 cells was washed with PBS, and macrophages were stimulated with LPS (50 ng/mL) for 3 h.

### 2.4. Microarray Analysis

The isolation of total RNA from *L. plantarum*-treated and non-lactobacilli-treated control macrophages was performed with the RNeasy Mini Kit (Qiagen, Tokyo, Japan). Samples were treated with DNAse and the integrity of RNA molecules was assessed with the RNA 6000 Nano Kit and the Agilent 2100 Bioanalyzer (Agilent Technologies, Santa Clara, CA, USA). Then, the biosynthesis of complementary DNA was performed with 200 ng of RNA. The SurePrint G3 Mouse GE 8 × 60K Ver.2.0 Microarray (Agilent Technologies) was used for hybridization at Hokkaido System Science Co. Microarray scanning was performed with the Microarray Scanner from Agilent Technologies, while digitization was performed with Agilent Feature Extraction 10.7.3.1.

The normalization of data and the analysis of gene expression were performed with GeneSpring software version 13.1 (Agilent Technologies). Genes up- and down-regulated significantly in samples (stimulated with LPS or *L. plantarum* plus LPS) with respect to control samples (without LPS stimulation) were considered. Two criteria were used for the selection of genes with significant changes in transcript abundance: a cutoff in transcript abundance of at least 2-fold and a *t*-test *p* value of less than 0.05. The Limma package from Bioconductor in R software (version 3.2.5) was used for the statistical studies. The log_2_ ratio was used to express the results. Genes whose expressions were log_2_ > 1 and *p* < 0.05 were annotated using PANTHER 11.1 (pantherdb.org, accessed on 4 April 2022) and analyzed according to the Gene Ontology (GO) classification. Microarray data were submitted to NCBI-GEO under the accession number GSE213389.

### 2.5. Two-Step Real-Time Quantitative PCR

Two-step real-time quantitative PCR (qPCR) was performed to characterize the expression of selected genes in macrophages. Total RNA isolation was performed with TRIzol reagent (Invitrogen). The Quantitect reverse transcription (RT) kit (Qiagen, Tokyo, Japan) was used to obtain the complementary DNAs following the manufacturer’s recommendations. The Platinum SYBR green qPCR SuperMix UDG with ROX (Invitrogen) and the 7300 real-time PCR system (Applied Biosystems, Warrington, UK) were used for qPCR. Table S1 show the sequence of primers used in this work. The PCR cycling conditions were described previously [4]. The reaction mixtures contained 5 μL of sample cDNA and 15 μL of master mix, including primers. β-actin expression was used to normalize cDNA levels in the samples.

### 2.6. L. plantarum LOC1 Genome Analysis

The identification of *L. plantarum* LOC1 at genus and species levels was performed with PCR and pheS sequence analysis. Whole-genome DNA from *L. plantarum* LOC1 was prepared following the procedure described previously [17].

The Prokaryotic Genome Annotation Pipeline (PGAP) v4.8 was used to predict bacterial genes. For this purpose, the stand-alone configuration was used. The functional characterization of individual genes in the LOC1 genome was performed with the BlastKOALA tool [19]. The analysis of glycosylhydrolases and glycosyltransferases was performed with the dbCAN2 server [20]. The amino acid sequences of extracellular proteins and adhesion factors as well as the proteins involved in the biosynthesis of EPSs, lipoproteins, and teichoic acids were obtained from the GenBank database. BLAST in the stand-alone mode [21], was used to search those genes in the genomes of *L. plantarum*. Figures showing the presence/absence of genes were performed with Python 3.7 using the Pandas and Seaborn libraries.

### 2.7. Statistical Analysis

Statistical analyses were performed using GLM and REG procedures available in the SAS computer program (SAS, 1994). Comparisons between mean values were carried out using one-way ANOVA and Fisher’s least significant difference (LSD) test. For these analyses, *p* values < 0.05 were considered significant.

## 3. Results

### 3.1. L. plantarum Isolated from Fresh Tea Leaves Modulate the Immunotranscriptomic Response of Macrophages Triggered by TLR4 Activation

**Figure 1:**
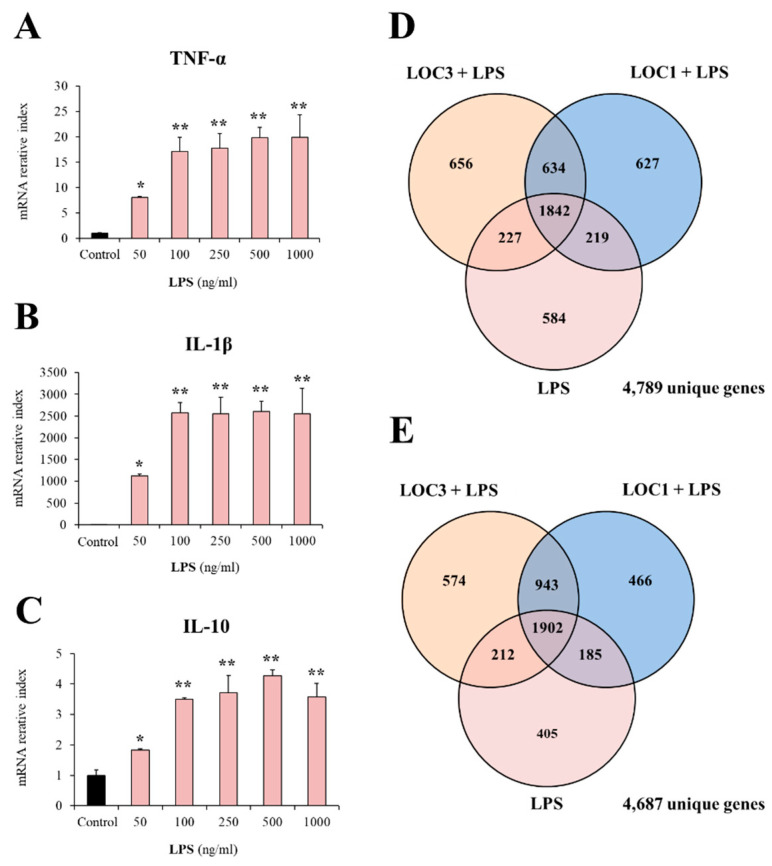
Effect of *Lactiplantibacillus plantarum* strains isolated from fresh tea leaves in murine RAW macrophages stimulated with the Toll-like receptor 4 (TLR4) agonist lipopolysaccharide (LPS). The expression of the immune factors *TNF-α* (**A**), *IL-β* (**B**) and *IL-10* (**C**) was determined 3 h after different doses of LPS stimulation. Asterisks indicate significant differences between the indicated groups and control macrophages, (*) *p* < 0.05, (**) *p* < 0.01. Macrophages were treated with *L. plantarum* LOC1 or LOC3 strains and then challenged with LPS. The expression of genes was determined by microarray analysis 3 h after LPS stimulation. Non-lactobacilli-treated macrophages stimulated with LPS were used as control. Venn diagrams showing the number of differentially up-regulated (**D**) and down-regulated (**E**) genes for each experimental group.

We first aimed to evaluate the response of RAW macrophages to the activation of TLR4 with different doses of LPS. For this purpose, macrophages were stimulated with 50, 100, 250, 500 and 1000 ng/mL of LPS and 3 h later, the expressions of *TNF-α*, *IL-1β* and *IL-10* were evaluated by qPCR (Figure 1A–C). The stimulation of macrophages with 50 ng/mL of LPS significantly augmented the expression of *TNF-α*, *IL-1β* and *IL-10.* The increase of 2 folds in LPS concentration (100 ng/mL) duplicated the expression levels of the three cytokines evaluated when compared to the lower dose of LPS (Figure 1A–C). Of note, further increases in LPS concentrations did not modify the expression levels of *TNF-α*, *IL-1β* or *IL-10* compared to the observed in macrophages stimulated with 100 ng/mL. Then, the dose of 100 ng/mL of LPS was selected for further experiments.

**Figure 2:**
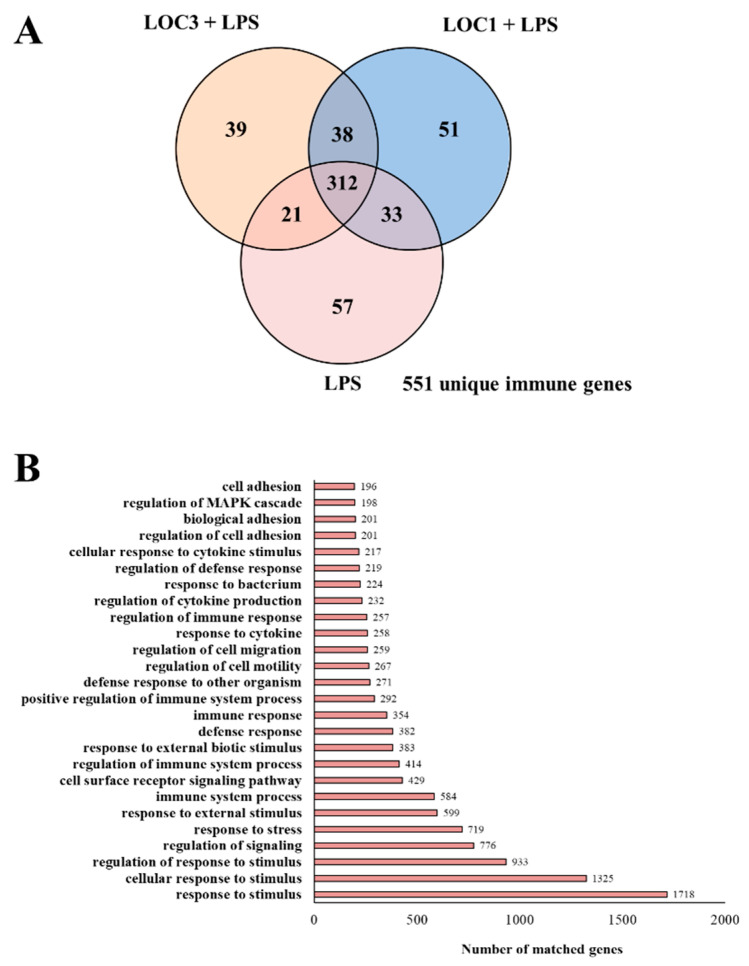
Effect of *Lactiplantibacillus plantarum* strains isolated from fresh tea leaves in murine RAW macrophages stimulated with the Toll-like receptor 4 (TLR4) agonist lipopolysaccharide (LPS). Macrophages were treated with *L. plantarum* LOC1 or LOC3 strains and then challenged with LPS. The expression of genes was determined by microarray analysis 3 h after LPS stimulation. Non-lactobacilli-treated macrophages stimulated with LPS were used as control. Venn diagrams showing the number of differentially regulated immune (**A**) genes for each experimental group. Number of matched genes categorized according to the Gene Ontology (GO) database (**B**).

The transcriptomic response of RAW macrophages to the challenge with LPS and the influence of *L. plantarum* LOC1 and LOC3 strains on this response was then investigated. Microarray analysis was performed in macrophages 3 h after the stimulation with the TLR4 agonist. When LPS-treated macrophages were compared with unchallenged RAW cells it was found that there were 4789 unique genes (Figure 1D) and 4687 unique genes (Figure 1E) up-regulated and down-regulated, respectively. Out of these differentially regulated genes, 551 were assigned to immune-related functions according to the GO database (Figure 2A). Changes in the immunotranscriptome response in macrophages after LPS challenge included transcripts in the following GO Biological Process pathways: “response to stimulus”, “response to stress”, “immune system process”, “cell surface receptor signaling pathway”, “response to external stimulus”, “regulation of the immune system process”, and “defense response” (Figure 2B).

**Figure 3:**
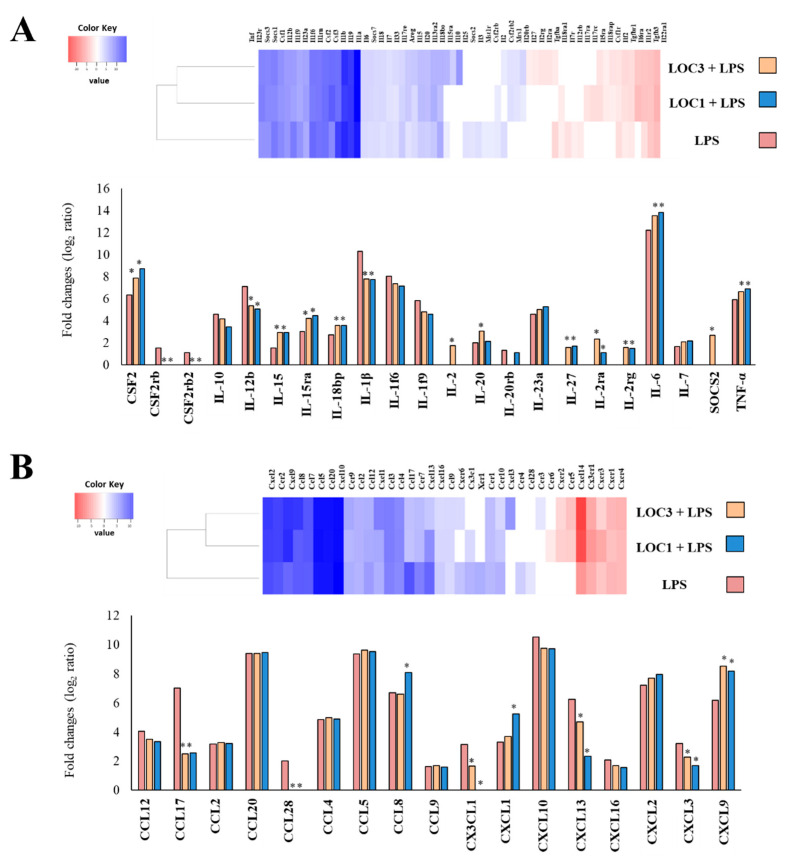
Effect of *Lactiplantibacillus plantarum* strains isolated from fresh tea leaves in murine RAW macrophages stimulated with the Toll-like receptor 4 (TLR4) agonist lipopolysaccharide (LPS). Macrophages were treated with *L. plantarum* LOC1 or LOC3 strains and then challenged with LPS. The expression of genes was determined by microarray analysis 3 h after LPS stimulation. Non-lactobacilli-treated macrophages stimulated with LPS were used as control. Heat-map analysis and fold expression changes of cytokines (**A**) and chemokines (**B**). Asterisks indicate significant differences between the indicated groups and LPS-challenged control macrophages, (*) *p* < 0.05.

The most remarkable changes in macrophages after stimulation with LPS were found in the expression of cytokines, chemokines and surface markers genes. LPS challenge augmented the expression of the inflammatory cytokines *TNF-α* (log_2_ ratio 5.9), *IL-1β* (10.3), *IL-6* (12.2), *IL-12b* (7.1), *IL-15* (1.5), *IL-18bp* (2.7), and *IL-23a* (4.6) (Figure 3A). In addition, the microarray analysis revealed significantly increases in the expression levels of the factors *CSF2* (6.3), *CSF2rb* (1.5) and *CSF2rb2* (1.5) (Figure 3A) as well as in the chemokines *CCL2* (3.1), *CCL4* (4.8), *CCL5* (9.3), *CCL8* (6.7), *CCL9* (1.3), *CCL12* (4.1), *CCL17* (7.1), *CCL20* (9.3), *CCL28* (2.1), *CX3CL1* (3.1), *CXCL1* (3.3), *CXCL2* (7.2), *CXCL3* (3.2), *CXCL9* (6.1), *CXCL10* (10.5), *CXCL12* (7.2), *CXCL13* (6.2), and *CXCL16* (2.1) (Figure 3B). Of note, augmented expression of the regulatory cytokine *IL-10* (4.6) was also detected in LPS-challenged macrophages (Figure 3A). Significantly increased expression of *CD40* (5.7), *CD69* (5.6), *CD80* (3.2), *CD83* (6.8), *CD164* (2.6), *CD200r1* (2.5), *Fcgr1* (2.1), *Fcgr4* (2.1), *Siglec1* (2.6) and *SiglecF* (3.3) were observed in RAW macrophages after the activation of TLR4 (Figure S1). In addition, the stimulation of macrophages with LPS increased the expression of *IFNA7* (1.2) and *IFNB1* (8.6) as well as several interferon-induced genes including *OAS1a* (2.5), *OAS2* (4.6), *OAS3* (3.5), *DXH58* (2.7), *IFIT3* (9.7), *IFI209* (7.5), *IFIT1b11* (6.5), and *IFIT3b* (9.5) (Figure S1).

Next, we analyzed microarray data to evaluate the effect of the strains *L. plantarum* LOC1 and LOC3 on the immunotranscriptomic response of macrophages stimulated with LPS. The comparative analysis of microarray profiles indicated that both LOC1 and LOC3 strains differentially modulated the expression of several genes related to the innate immune response triggered by TLR4 activation in macrophages. The Venn diagram analysis was used to find genes that were uniquely and commonly modulated between *L. plantarum*-treated and control macrophages (Figure 2A). Of the 551 differentially expressed genes in the Venn diagram analysis, 51 were unique to the cells stimulated with the LOC1 strain before the LPS challenge, while 39 were unique for the LOC3 group. In addition, 38 genes were common to LOC1 treatment plus LPS and LOC3 plus LPS groups. It was also observed that 312 genes were common to all the three treatments (Figure 2A).

The heat-map cluster analysis focused on the expression of cytokines, chemokines (Figure 3), surface markers and IFN-related genes (Figure S1) depicts the transcriptomic patterns of differentially modulated genes between *L. plantarum*-treated and control macrophages. The treatment with LOC1 plus LPS clustered closer to the treatment with LOC3 plus LPS and, both clustered separated from the control. Closer examination of gene expression revealed differences in cytokines and chemokines genes sheared by *L. plantarum*-treated macrophages and controls (Figure 3). Most remarkable differences were found in the genes for *CSF2*, *IL-6*, *TNF-α*, *IL-15* and *IL-18bp* that were significantly higher in lactobacilli-treated macrophages than in controls. In addition, the expression levels of *IL-1β* and *IL-12b* were lower in macrophages treated with the LOC strains than in controls (Figure 3A). Interestingly, although no differences were found between *L. plantarum*-treated macrophages and controls when the expression of *IL-10* was analyzed, it was detected that macrophages stimulated with the LOC1 strain significantly up-regulated the expression of the regulatory factors *IL-27* and *SOCS2*. *L. plantarum* LOC3 was able to increase the expression of *IL-27* but not *SOCS2* (Figure 3A). The LOC strains were also able to significantly reduce the expressions of *CCL17*, *CCL28*, *CX3CL1*, *CXCL13* and *CXCL3*, being the LOC1 strain more efficient than the LOC3 to reduce the expression levels of *CXCCL1* and *CXCL13* (Figure 3B). Both *L. plantarum* strains increased the expression of *CXCL9*, while only the LOC3 strain augmented the expression of *CCL8* and *CXCL1* when compared to controls (Figure 3B). The LOC strains reduced the expression of *CD38*, *CD83*, *CD164*, *CD200r2*, *CD209a*, and *SiglecF* in macrophages challenged with LPS, while they augmented the expressions of *CD200r4*, *CD80*, *CD86*, *H2-K1* and *H2-M3* (Figure S1). Only *L. plantarum* LOC1 increased the expression of *CD200b*, while only LOC3 augmented *Siglec1* (Figure S1). When the IFN-related genes were analyzed, it was observed that both *L. plantarum* LOC1 and LOC3 significantly increased the expression of *IFNB1*, *IFNA7*, *IFIT3*, *IFIT1b11*, and *IFIT3b* (Figure S1). Furthermore, significantly up-regulated expression of *IFNA1*, *IFNA2*, *IFNA5*, *IFNA13*, *IFNA14*, *IFITM7*, and *IFNAB* was detected in macrophages treated with the LOC strains when compared to controls (Figure S1).

### 3.2. L. plantarum LOC1 Modulate the TLR4-Mediated Immune Response in Macrophages Similarly to Other Immunobiotic Strains

**Figure 4:**
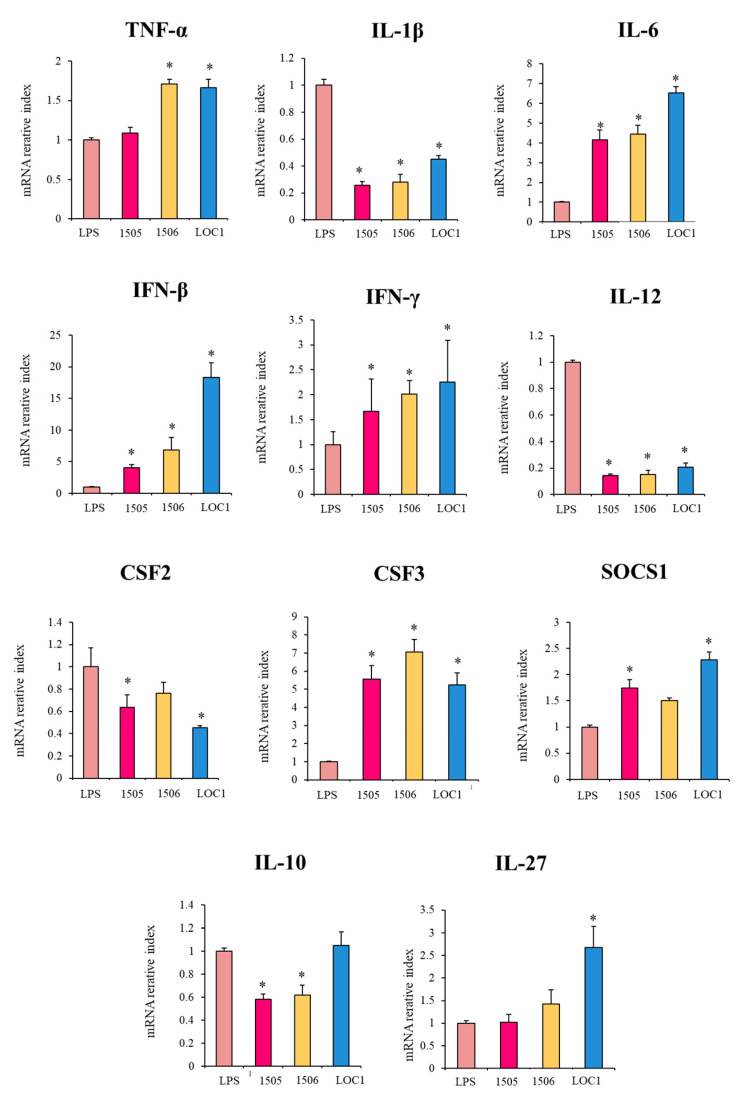
Effect of *Lactiplantibacillus plantarum* LOC1 in murine RAW macrophages stimulated with the Toll-like receptor 4 (TLR4) agonist lipopolysaccharide (LPS). Macrophages were treated with *L. plantarum* LOC1 and then challenged with LPS. The expression of immune factors was determined 3 h after LPS stimulation. Macrophages treated with the probiotic strains *Lacticaseibacillus rhamnosus* CRL1505 or *L. plantarum* CRL1506 and then challenged with LPS were used for comparisons. Results represent data from three independents. Asterisks indicate significant differences between the indicated groups and LPS-challenged control macrophages, (*) *p* < 0.05.

In order to confirm the changes induced by *L. plantarum* LOC1 in the immunotranscriptome response of LPS-challenged macrophages, qPCR was performed on selected genes. Genes with significant differences between LOC1-treated and non-treated macrophages were chosen. The expression of IL-10 was also evaluated. In addition, the influence of the well-characterized immunobiotic strains *L. rhamnosus* CRL1505 [22] and *L. plantarum* CRL1506 [23] on the response of macrophages to TLR4 activation was also studied for comparisons. As shown in Figure 4, the transcriptional changes evaluated by qPCR indicated a similar overall trend in the transcription of microarray. The treatment of macrophages with *L. plantarum* LOC1 significantly increased their expression levels of *TNF-α*, *IL-6*, *IFN-β*, *IFN-γ*, and *CSF3* in response to LPS challenge. The LOC1 strain also reduced the expression of *IL-1β*, *IL-12*, and *CSF2* in macrophages stimulated with the TLR4 agonist. Similarly, the treatment of macrophages with the CRL1505 and CRL1506 strains significantly augmented the expressions of *IL-6*, *IFN-β*, *IFN-γ*, and *CSF3* and reduced *IL-1β* and *IL-12* (Figure 4). No significant reductions were observed for *TNF-α* and *CSF2* in macrophages treated with *L. rhamnosus* CRL1505 and *L. plantarum* CRL1506, respectively. Of note, *L. plantarum* LOC1 was more efficient than CRL1505 and CRL1506 strains to increase the expression of *IFN-β* (Figure 4). As expected, the LOC1 strain did not induce changes in *IL-10* expression, while both CRL1505 and CRL1506 significantly reduced the expression levels of the regulatory cytokine. In addition, *L. rhamnosus* CRL1505 and *L. plantarum* LOC1 enhanced the expression of *SOCS1*, while only the LOC1 strain up-regulated *IL-27* when compared to LPS-challenged macrophages (Figure 4).

**Figure 5:**
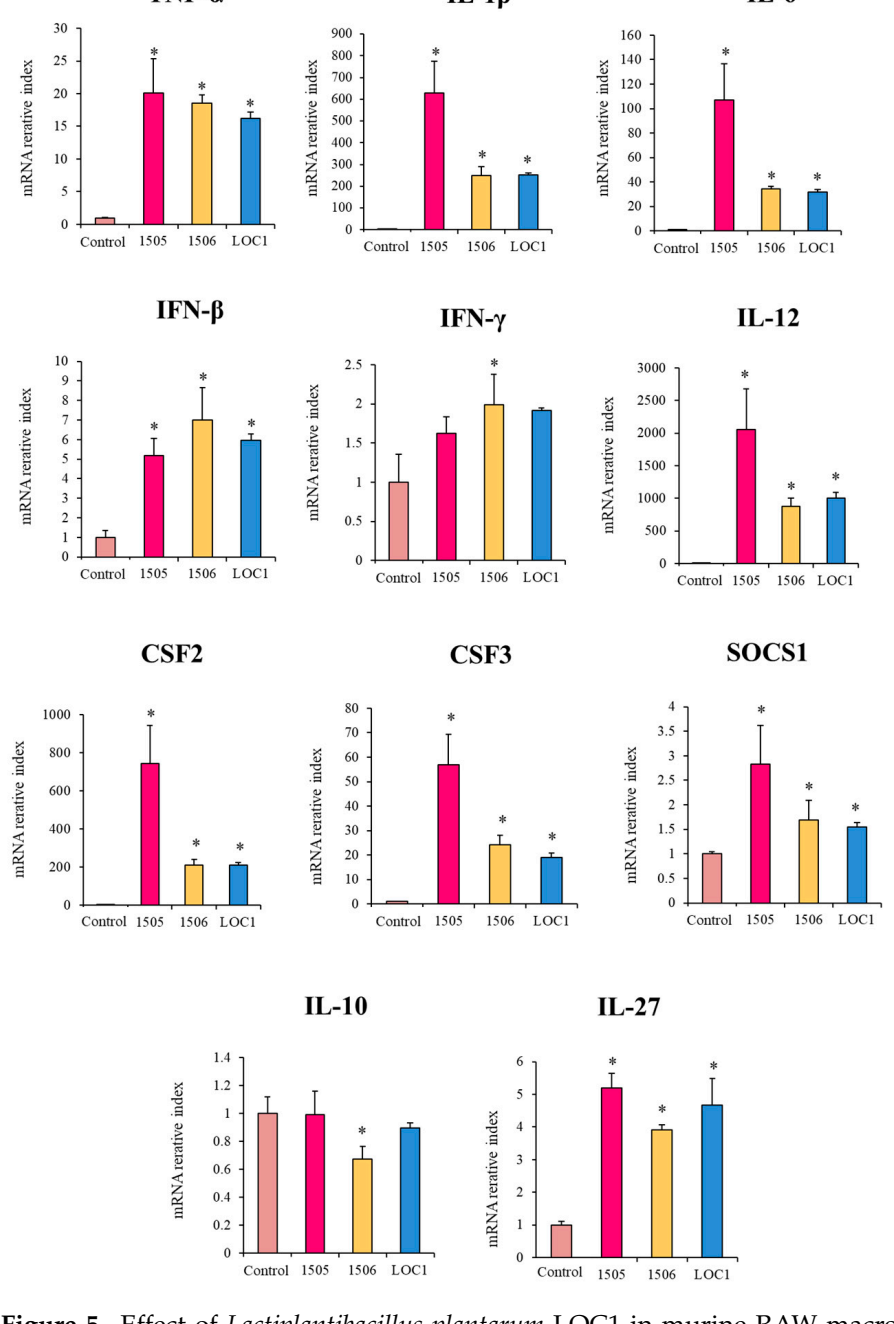
Effect of *Lactiplantibacillus plantarum* LOC1 in murine RAW macrophages. Cells were treated with *L. plantarum* LOC1 and the expression of immune factors was determined 12 h after lactobacilli stimulation. Macrophages treated with the probiotic strains *Lacticaseibacillus rhamnosus* CRL1505 or *L. plantarum* CRL1506 were used for comparisons. Results represent data from three independents. Asterisks indicate significant differences between the indicated groups and untreated control macrophages, (*) *p* < 0.05.

We also evaluated the effect of lactobacilli in the expression of selected immune factors in macrophages in the absence of LPS challenge. As shown in Figure 5, the three strains increased the expression of *TNF-α*, *IL-1β*, *IL-6*, *IFN-β*, *IL-12*, *CSF2* and *CSF3* in macrophages when compared to untreated controls. The three strains were equally effective in up-regulating *TNF-α* and *IFN-β* expressions, while the levels of *IL-1β*, *IL-6*, *IL-12*, *CSF2* and *CSF3* were significantly higher in macrophages treated with *L. rhamnosus* CRL1505 than in cells stimulated with the *L. plantarum* strains. In addition, the CRL1506 strain was the only one able to increase the expression of *IFN-γ* (Figure 5). The three strains augmented the expression of *SOCS1* and *IL-27*. The lactobacilli treatment did not induce modifications in the expression levels of *IL-10* except for *L. plantarum* CRL1506 that reduced this regulatory cytokine (Figure 5).

### 3.3. L. plantarum LOC1 Does Not Modulate the TLR4-Mediated Immune Response in Macrophages Indirectly Througth Intestinal Epithelial Cells

**Figure 6:**
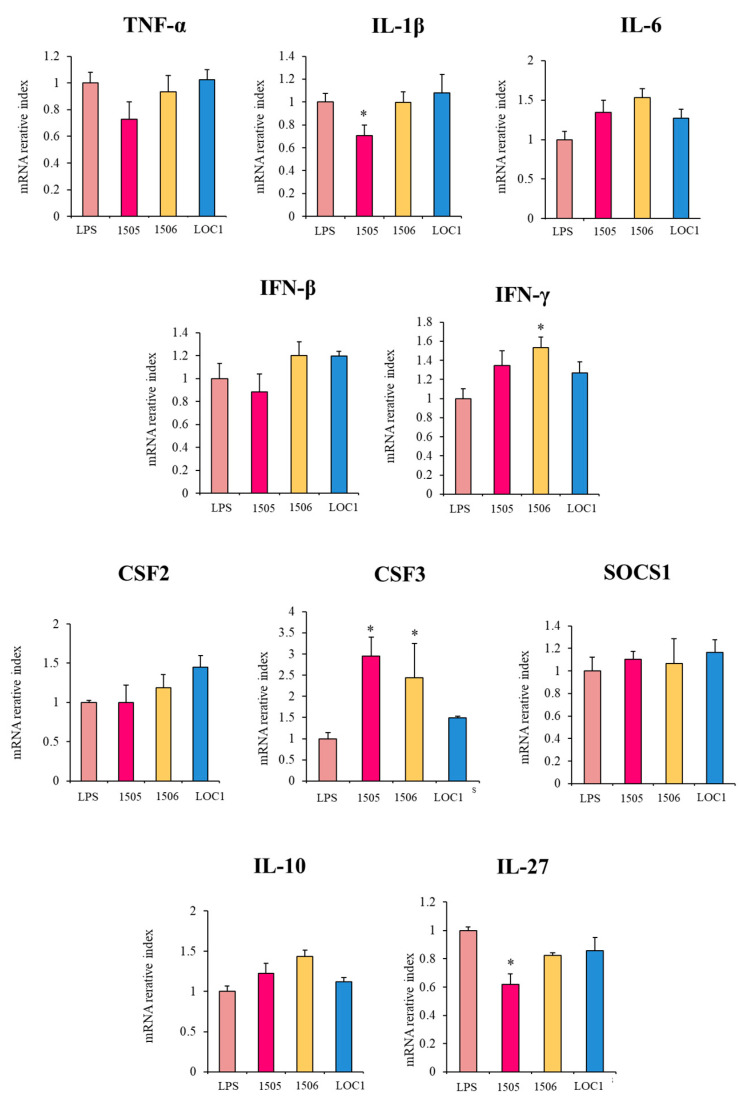
Effect of *Lactiplantibacillus plantarum* LOC1 in murine intestinal epithelial (MIE) cells and RAW macrophage co-cultures stimulated with the Toll-like receptor 4 (TLR4) agonist lipopolysaccharide (LPS). MIE cells in co-culture with macrophages were treated with *L. plantarum* LOC1 and then challenged with LPS. The expression of immune factors was determined in macrophages 3 h after LPS stimulation. Co-cultures treated with the probiotic strains *Lacticaseibacillus rhamnosus* CRL1505 or *L. plantarum* CRL1506 and then challenged with LPS were used for comparisons. Results represent data from three independents. Asterisks indicate significant differences between the indicated groups and LPS-challenged control co-cultures, (*) *p* < 0.05.

It was demonstrated that intestinal epithelial cells play a crucial role in the interaction of the intestinal immune system with microbes. Epithelial cells influence immune responses by orchestrating communication between intestinal microbes and mucosal innate immune cells such as macrophages [24]. Thus, we aimed to evaluate whether *L. plantarum* LOC1, *L. rhamnosus* CRL1505 or *L. plantarum* CRL1506 were able to influence the response of macrophages to LPS challenge indirectly through intestinal epithelial cells. For this purpose, murine intestinal epitheliocytes (MIE cells) co-cultures with RAW macrophages were prepared. MIE cells were stimulated with LOC1, CRL1505 or CRL1506 strains and then macrophages were challenged with the TLR4 agonist, and the expressions of immune factors were evaluated by qPCR (Figure 6). No significant differences were observed between control and lactobacilli-treated cells when the expressions of *TNF-α*, *IL-6*, *IFN-β*, *CSF2*, *SOCS1* and *IL-10* were evaluated. The treatment with *L. rhamnosus* CRL1505 reduced the expression of *IL-1β* and *IL-27*, while the CRL1506 improved *IFN-γ* (Figure 6). Both, *L. rhamnosus* CRL1505 and *L. plantarum* CRL1506 significantly increased the expression levels of *CSF3*. Of note, the treatment with *L. plantarum* LOC1 did not induce changes in *TNF-α*, *IL-6*, *IFN-β*, *CSF2*, *CSF3*, *SOCS1* or *IL-10* (Figure 6).

### 3.4. General Genomic Characteristics of L. plantarum LOC1

We next aimed to carry out comparative and functional genomics studies in order to identify the bacterial gene(s) responsible for the immunomodulatory properties of *L. plantarum* LOC1. For this purpose, the complete genome of the LOC1 strain was sequenced with the Illumina MiSeq platform and uploaded to the NCBI repository (accession number BOUN00000000).

The whole genome size and GC content of *L. plantarum* LOC1 was 3.13 Mb and 44.7%, respectively (Table 1). This is in agreement with the genomic analysis of *L. plantarum* strains that showed a mean size and GC content of 3.32 Mb and 44.5%, respectively [25].

**Figure 7:**
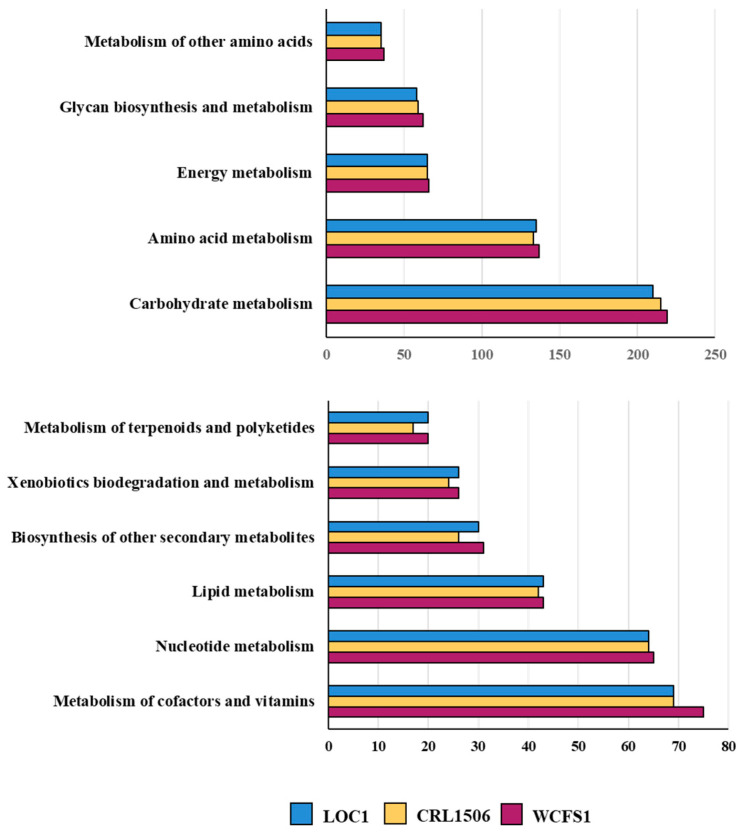
Study of the number of genes in the different functional categories associated with metabolic pathways of the genome of *Lactiplantibacillus plantarum* LOC1. The genome of the LOC1 strain was studied and compared with the probiotic strains WCFS1 and CRL1506 as reference. Functional characterization of genes was performed with the BlastKOALA tool according to the KEGG database.

The KEGG database and the BlastKOALA tool were used for the functional characterization of the LOC1 strain genome (Figure 7). *L. plantarum* CRL1506 and the well-characterized probiotic strain WCFS1 were used for comparisons. No differences were observed in the number of metabolism pathway genes associated with carbohydrates (213 ± 2), glycan biosynthesis and metabolism (59 ± 1), amino acids (135 ± 2), lipids (41 ± 2), nucleotides (65 ± 2) or energy metabolism (65 ± 2) among the strains. In addition, no significant differences were found in cofactors and vitamins (69 ± 3), polyketides (18 ± 3) or biosynthesis of other secondary metabolites (27 ± 3) when the genomes of LOC1, CRL1506 and WCFS1 were compared (Figure 7). There were also no differences in the numbers of genes associated with “genetic information processing”, “environmental information processing” or “cellular processes” between the three lactobacilli strains (Figure S2).

**Figure 8:**
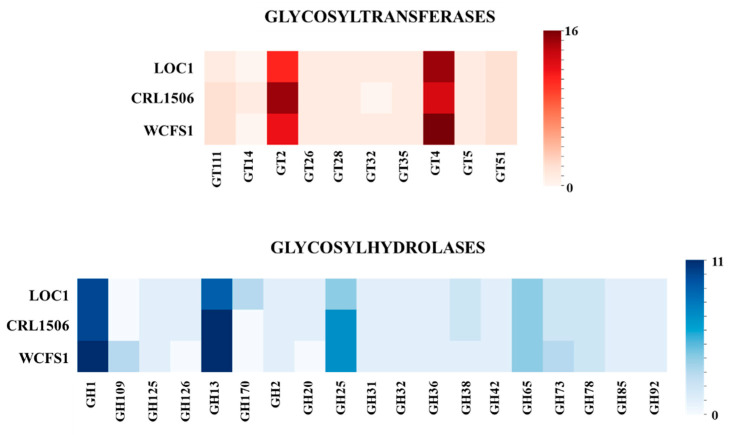
Analysis of the number of genes of the different families of glycosyltransferases (GT) and glycosylhydrolases (GH) in *Lactiplantibacillus plantarum* LOC1. The genome of the LOC1 strain was studied and compared with the probiotic strains WCFS1 and CRL1506 as reference.

Previous genomic analyzes found that in the species *L. plantarum* the most abundant sugar metabolism genes belonged to the glycosylhydrolases (GH) and glycosyltransferases (GT) families [26]. Thus, the numbers of genes for GT and GH were also analyzed in the LOC1 strain (Figure 8). Genes for several GT and GH families were detected in the genomes of *L. plantarum* LOC1. Similar to *L. plantarum* WCSF1 the LOC1 strain had numbers of GT2 and GT4 that were lower and higher than the CRL1506 strain, respectively. In addition, lower numbers of GH13 and GH25 were found in the LOC1 strain when compared to *L. plantarum* WCSF1 and CRL1506 (Figure 8). *L. plantarum* LOC1 had also higher numeber of GH170 than the other two strains. Similar to *L. plantarum* CRL1506, the LOC1 strain had numbers of GH1, GH109 and GH73 that were lower than the WCSF1 strain.

### 3.5. Study of the Genes Associated with the Expression of Surface Molecules in L. plantarum LOC1

**Figure 9:**
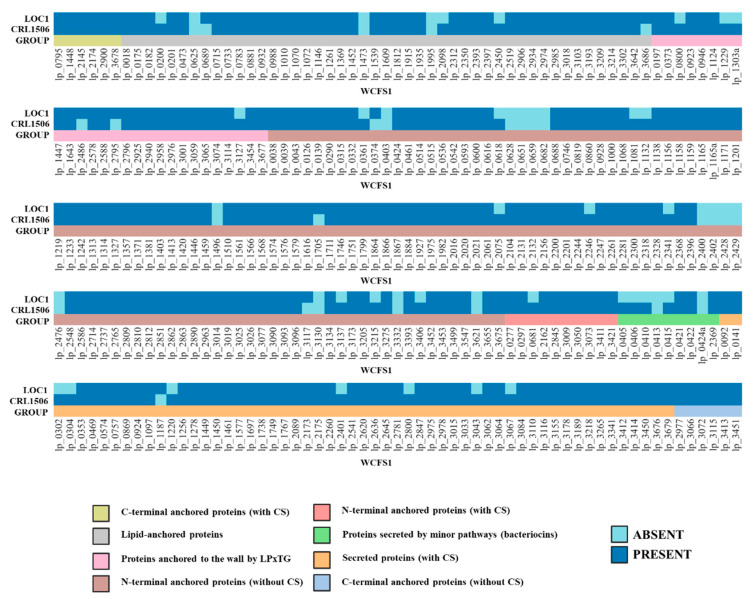
Analysis of the presence/absence of extracellular proteins in *Lactiplantibacillus plantarum* LOC1. The genomes of LOC1 and CRL1506 strains were studied and compared with the probiotic strain WCFS1 as reference (*lp_* genes). Proteins were grouped in C-terminal anchored proteins with and without cleavage sites (CS), N-terminal anchored proteins with and without CS, lipid-anchored proteins, proteins anchored to the wall by the LPxTG domain, secreted proteins with CS and proteins secreted by minor pathways.

It was reported that surface-exposed proteins play key roles in the interactions of lactobacilli with their environments [27,28]. Therefore, we first evaluated the genes coding surface-exposed and secreted proteins in *L. plantarum* LOC1 and compared them with CRL1506 and WCSF1 strains (Figure 9). The extracellular proteins in the LOC1 strain were studied by in silico analysis considering the subcellular location (surface-expressed or secreted), the secretory mechanism and the anchorage type, following the guidelines recently described by our group [28]. Notable differences were detected between *L. plantarum* LOC1, CRL1506 and WCSF1 when analyzing the 304 genes for extracellular proteins. Of note, the proteins ABC transporter-substrate binding protein (lp_0200), prophage P2a protein 7 (lp_2450), cell surface protein precursor with LPXTG-motif (lp_0800), mucus-binding protein with LPXTG-motif cell wall anchor (lp_3127), preprotein translocase SecE (lp_0616), extracellular protein (lp_1132), transcriptional attenuator LytR family (lp_2075), bacterial type II secretion/trafficking system extracellular protein (lp_2246), class A beta-lactamase (lp_2341), PTS system EIIB component (lp_3137), zinc ribbon domain-containing protein (lp_3215), membrane-bound cell surface hydrolase (lp_3393), prophage P1 lysin (lp_0681), bacteriocin precursor peptide PlnK (lp_0405), bacteriocin precursor peptide PlnJ (lp_0406), bacteriocin precursor peptide PlnN (lp_0410), plantaricin A precursor induction factor (lp_0415), extracellular transglycosylase (lp_0302), extracellular transglycosylase (lp_0304), polysaccharide biosynthesis protein (lp_1220), prophage P2a lysin (lp_2401), transcription regulator MarR (lp_2800), extracellular zinc metalloproteinase (lp_3043), and the cell surface protein CscB (lp_3067) that are present in the genomes of the CRL1506 and WCSF1 strains were absent in *L. plantarum* LOC1 (Figure 9). In addition, the proteins cell surface lipoprotein precursor (lp_0689), ABC transporter-substrate binding protein (lp_3686), mucus-binding protein with LPXTG-motif cell wall anchor (lp_2486), cell surface protein precursor (lp_2795), lysine-rich extracellular protein (lp_0374), cell surface protein CscC (lp_3117) and glycosylhydrolase (lp_1187) were detected in the genome of the LOC1 strain but not in *L. plantarum* CRL1506 (Figure 9).

**Figure 10:**
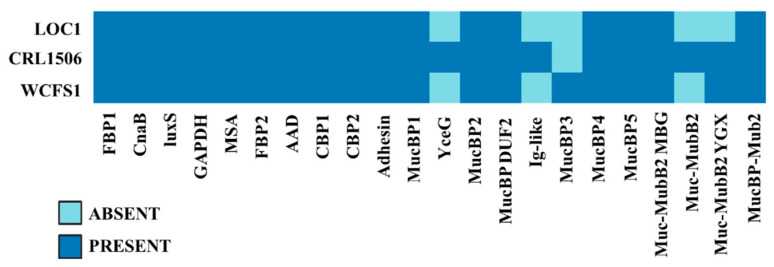
Analysis of the presence/absence of genes involved in adhesion in *Lactiplantibacillus plantarum* LOC1. The genome of the LOC1 strain was studied and compared with the probiotic strains WCFS1 and CRL1506 as reference.

We also investigated the presence of genes involved in the adhesion to the gastrointestinal tract in the genome of *L. plantarum* LOC1. For this purpose, a bioinformatic analysis was carried out considering different types of molecules with mucus/mucin-binding domains, which were recently characterized and described in a comparative genomic study using strains of *L. plantarum* possessing differential immunomodulatory activities [28]. Then, the proteins MucBP1, MucBP2, MucBP3, MucBP4, MucBP5, MucBP-DUF1, MucBP-DUF2, Muc-MubB2-MBG, Muc-MubB2-YGX, Muc-MubB2, YceG and Ig-like were searched in the genome of the LOC1 strain (Figure 10). Similar to the WCSF1 strain, *L. plantarum* LOC1 does not possess the proteins YceG, Ig-like, and Muc-MubB2. The proteins MucBP3 and Muc-MubB2-YGX were also absent in the genome of the LOC1 strain.

Proteins containing domains for chitin, collagen, and fibronectin adhesion have been described in the genome of *L. plantarum* WCFS1 [29,30,31,32]. Then, the presence of the genes coding for collagen binding proteins CBP1 and CBP2, fibronectin binding proteins FBP1 and FBP2, as well as Msa (mannose-specific binding), GAPDH (glyceraldehyde-3-phosphate dehydrogenase), LuxS (autoinducer-2), AAD (alpha-acetolactate decarboxylase) and CnaB (Cna protein type B) proteins were searched in the genome of the LOC1 strain and compared with *L. plantarum* CRL1506 and WCFS1 (Figure 9). All these adhesion factors were detected in the genome of the LOC1 strain.

**Figure 11:**
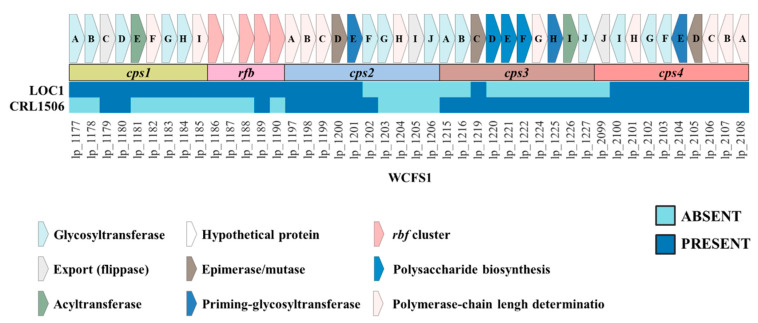
Analysis of the presence/absence of genes involved in the biosynthesis of exopolysaccharides (EPS) in *Lactiplantibacillus plantarum* LOC1. The genomes of LOC1 and CRL1506 strains were studied and compared with the probiotic strain WCFS1 as reference (*lp_* genes). Four clusters for EPS biosynthesis (cps1, cps2, cps3 and cps4) and the *rfb* cluster involved in the incorporation of rhamnose to EPS are shown.

EPSs are also involved in the interaction of LAB with the host. It was shown that *L. plantarum* have four clusters for the biosynthesis of EPS [33] and recently we demonstrated that strains may differ markedly in the presence of those clusters in their genomes [28]. Thus, we performed a sequence comparison using *L. plantarum* WCFS1 as a reference to characterize the EPS clusters in the genome of the LOC1 strain (Figure 11). The genes corresponding to the eps1 cluster (or cps1) and eps4 cluster (or cps4) were found in the *L. plantarum* LOC1 except for the *eps4J* gene of cluster eps4. The genes corresponding to the eps3 cluster (or cps3) were not found in the genomes of any of the LOC1 strain, except for the *eps3C* gene (Figure 11). For the eps2 (or cps2) cluster, it was observed that *L. plantarum* LOC1 had the first five genes (*eps2A*, *eps2B*, *eps2C*, *eps2D* and *eps2E*) that have been reported to be highly conserved among *L. plantarum* strains [33,34]. The other genes of the eps2 cluster of the WCSF1 strain used as reference were not found in *L. plantarum* LOC1 and this is probably related to the fact that those genes possess very limited sequence homology between the corresponding regions from different strains [28,34]. It was shown that some strains including *L. plantarum* WCFS1 [34] possess a group of conserved genes called *rfb* (*rmlACBD* genes), which are involved the biosynthesis of rhamnose precursors during the synthesis of EPS molecules [35]. The *rfb* genes were detected in the genome of *L. plantarum* LOC1 (Figure 11).

Some studies have demonstrated that lipoproteins expressed on the surface of LAB may be involved in their immunomodulatory effect. In this regard, it was shown that the prolipoprotein diacylglyceryl transferase *lgt* gene (lp_0755), is of important for the immunomodulatory ability of *L. plantarum* WCFS1 [36]. Furthermore, it was shown that the WCFS1 possess three acyltransferases (lp_0856, lp_0925 and lp_1181) that are involved in lipoprotein triacylation. On the other hand, it was reported that teichoic (TA) and lipoteichoic (LTA) acids, and particularly the *dltD* gene involved in the incorporation of D-alanine into LTA, play important roles in the immunomodulatory capacities of probiotic *L. plantarum* strains [12,37,38,39]. Then, we investigated the presence of *lgt*, lp_0856, lp_0925, lp_1181 and *dltD* genes as well as other genes involved in TA and LTA biosynthesis in the genome of the LOC1 strain (Figure S3). All the genes were present in *L. plantarum* LOC1 and showed no differences with WCSF1 and CRL1506 strains, except for *tagF1* (lp_0268) and *tagF2* (lp_0269).

## 4. Discussion

Macrophages represent the first line of host immune defense in the intestine [40,41] and their proper activity requires a tight regulation of gene expression allowing a fine-tuned immune response. It was reported that probiotic microorganisms are able to beneficially modulate macrophage functioning by enhancing their ability to protect against pathogens and avoiding the generation of detrimental inflammation (reviewed in Wang et al. [42]). Of note, the ability of probiotic bacteria to modulate macrophages’ responses was shown to be strain specific and thus, each probiotic candidate must be tested to identify specific biological activities. The murine RAW 264.7 macrophage cell line has been shown to be a useful in vitro tool to select and characterize immunomodulatory probiotics [42]. Using RAW macrophages, we demonstrated in this work that *L. plantarum* LOC1 isolated from fresh tea modulates cytokine expressions. The LOC1 strain increased the expression of *TNF-α*, *IL-1β*, *IL-6*, *IFN-β*, *IL-12*, *CSF2* and *CSF3*. In addition, *L. plantarum* LOC1 did not induce modifications in *IL-10* but it augmented the expression of *SOCS1* and *IL-27*. The changes induced by the LOC1 strain were characteristic and differed from those induced by other probiotic strains such as *L. rhamnosus* CRL1505 and *L. plantarum* CRL1506, highlighting that this property is strain dependent. In line with our results, it was found that RAW macrophages treated with *L. acidophilus* MTCC-10307 significantly up-regulate the expression of *IL-10*, *IL-6*, *IL-12* and *IFN-α*, while the expression of *TNF-α* was down-regulated with low doses of lactobacilli but up-regulated at higher concentrations [43]. The treatment of RAW macrophages with *L. rhamnosus* ATCC 7469 increased the synthesis of TNF-α, IL-6, and IL-10, but was not able to enhance the production of IL-1β, IL-4 or IL-12 [44].

Our results suggest that *L. plantarum* LOC1 is a potent stimulator of macrophages. The improvement of *TNF-α*, *IL-1β*, *IL-6*, and *IL-12* have been shown to be associated with the enhanced capacity of macrophages to protect against infections. Studies found that lactobacilli with the ability to reduce the severity of intestinal infections can improve the phagocytic activity of macrophages and their capacity to produce inflammatory factors such as TNF-α and IFN-γ [45,46]. It was also reported that immunostimulatory *L. plantarum* strains activate mouse macrophages enhancing IL-12 production and phagocytosis [47]. Furthermore, studies demonstrated that among the cytokines induced by immunomodulatory lactobacilli, the most remarkable effect was the increase in TNF-α, IFN-γ, IL-1β, IL-6, and IL-12 for most of the probiotic strains assayed [45,46,48]. Of note, the LOC1 strain also increased *SOCS1* and *IL-27* that are known to be involved in the control of inflammation [49,50]. Thus, the LOC1 strain is capable of modulating both the inflammatory and regulatory responses in macrophages. This property was put into further evidence when *L. plantarum* LOC1-treated macrophages were challenged with LPS.

**Figure 12:**
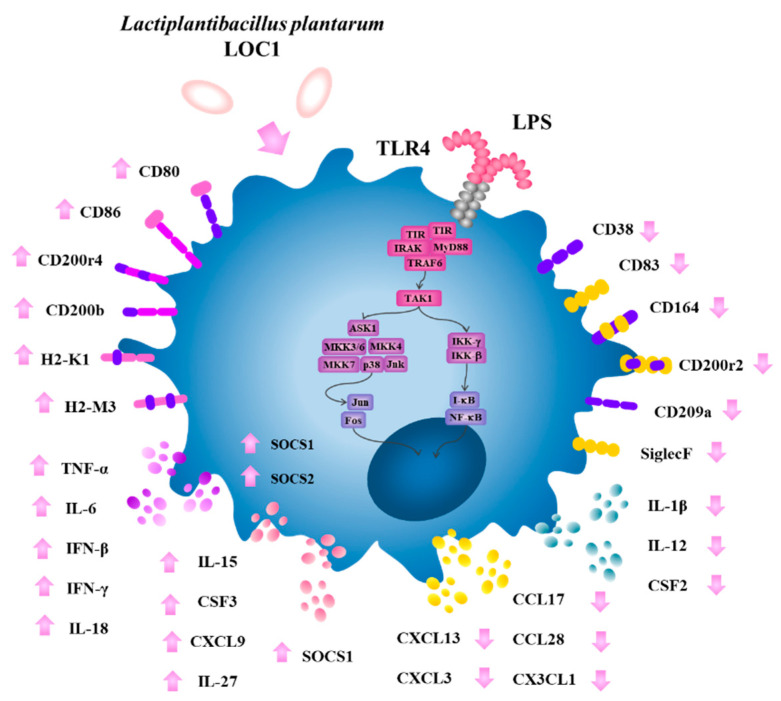
Schematic representation of the immune factor expressions differentially by *Lactiplantibacillus plantarum* LOC1 in murine RAW macrophages stimulated with the Toll-like receptor 4 (TLR4) agonist lipopolysaccharide (LPS). Up- and down-regulation of immune genes with respect to LPS-treated control macrophages are shown with arrows.

Several large-scale transcriptomic and proteomic studies have been performed with macrophages challenged with LPS with the aim of exploring different aspects of the TLR4 signaling and its impact on mucosal protection [40,41]. In this work, we carried out a transcriptomic study to evaluate the response of murine macrophages to the activation of TLR4 and in line with several other studies [51,52], we found that RAW macrophages mount a complex transcriptomic response characterized by changes in the expression of hundreds of genes with diverse immunological functions. Of note, the most remarkable changes in RAW macrophages after the stimulation with LPS were found in expression of cytokines, chemokines and surface markers genes. Using this transcriptomic approach, we also demonstrated that *L. plantarum* LOC1 exerts a modulatory effect on LPS-induced inflammation, resulting in a differential regulation of immune factor expression in macrophages. Specifically, we found that the LOC1 strain markedly reduced the LPS-induced expression of some inflammatory cytokines (*IL-1β*, *IL-12*, and *CSF2*) and chemokines (*CCL17*, *CCL28*, *CXCL3*, *CXCL13*, *CXCL1*, and *CX3CL1*), while it significantly increased the expression of other cytokines (*TNF-α*, *IL-6*, *IL-18*, *IFN-β*, *IFN-γ*, and *CSF3*), chemokines (*IL-15* and *CXCL9*), and activation markers (*H2-k1, H2-M3, CD80,* and *CD86*) in RAW macrophages (Figure 12).

The cytokines TNF-α, IL-6, IL-18, IFN-β, IFN-γ, and CSF3 produced by macrophages play important roles in the protection again pathogens by promoting phagocytosis and mediating the recruitment and activation of effector immune cells into the site of infection. These functions are complemented by chemokines such as IL-15 and CXCL9 that induce the recruitment of γδ T cells [53] and T cells [54], respectively, which are essential for the protection against Gram negative pathogens. In addition to cytokine and chemokine up-regulation, macrophages increase several activation markers after their stimulation with LPS. In this regard, although MHC class II molecules are constitutively expressed on macrophages, its expression is significantly augmented by LPS [55]. We observed a significant increase in several histocompatibility class II antigens in RAW macrophages challenged with LPS. Of note, *L. plantarum* LOC1 significantly up-regulated the expression of histocompatibility 2, class II antigen K1 and histocompatibility 2, class II antigen M3 as well as the co-stimulatory molecules CD80 and CD86 (Figure 12). MHC class II and co-stimulatory proteins expressed in macrophages are of importance in the development and maintenance of immune responses mediated by T cells, particularly CD4^+^ T lymphocytes [55]. Thus, our results show that the LOC1 strain is able to stimulate macrophages to support T-cell-mediated immunity.

Of note, we also detected the reduction in inflammatory factor expressions in LPS-treated macrophages previously stimulated with *L. plantarum* LOC1 (Figure 12). The reduction in inflammatory cytokines expression in LPS-challenged RAW macrophages have been described for other LAB strains. It was shown that *L. plantarum* CKDB008 reduce the production of NO, TNF-α, IL-6, and IL-1β in LPS-stimulated RAW macrophages [56]. Lactobacilli strains isolated from different sources reduced the levels of TNF-α, IL-1β and IL-6 by inhibiting p38, ERK1/2 and SAPK/JNK pathways in RAW cells challenged with LPS [57]. Similarly, cellular fractions derived from the probiotic strain *L. rhamnosus* GG were shown to inhibit the activation of MAPK and NF-κB signaling pathways in LPS-stimulated RAW cells, leading to reduced TNF-α and IL-6 production [58]. Here, the treatment of macrophages with the LOC1 strain significantly reduced the expression of *IL-1β*, *IL-12*, and *CSF2* as well as *CCL17*, *CXCL3*, *CXCL13*, *CX3CL1* and *CXCL1*, which are chemokines for neutrophils, monocytes, macrophages, and B cells [55]. We also detected the up-regulation of *SOCS-1* that is a negative regulator able to control cytokine signaling [49] and *IL-27* that is an important regulator of inflammation that limit development of IFNγ-producing Th1 cells by stimulating IL-10 production by CD4^+^ T cells [50]. Furthermore, our transcriptomic analysis revealed a differential expression of CD200/CD200R molecules, which reduce the synthesis of proinflammatory mediators and induces the synthesis of anti-inflammatory factors in macrophages [59]. A reduction in the pro-inflammatory marker CD38 [60] was also observed in macrophages treated with the LOC1 strain.

In the presence of inflammatory stimuli such as LPS, macrophages change their gene expression, up-regulating proinflammatory cytokines and chemokines, oxidative metabolites, and proteases that play roles in the protection against pathogens. However, if the macrophage-mediated inflammatory response is not timely and effectively regulated, tissue-destructive pathology is induced [41]. Macrophages show considerable phenotype diversity and plasticity in response to environmental stimuli and can behave as both inflammatory and regulatory cells. Our results allow us to speculate that *L. plantarum* LOC1 would enhance the intrinsic functions of macrophages, promoting their protective effects mediated by the stimulation of the Th1 response without affecting the regulatory mechanisms that help control inflammation. Whether this effect is maintained in vivo, helping to improve protection against pathogens and protecting against inflammation simultaneously, is a topic that should be studied in the immediate future to position this strain as a probiotic to improve resistance to infections. In addition, detailed mechanistic studies are necessary to evaluate the immune receptor(s) used by *L. plantarum* LOC1 to exert its immunomodulatory effects.

**Figure 13:**
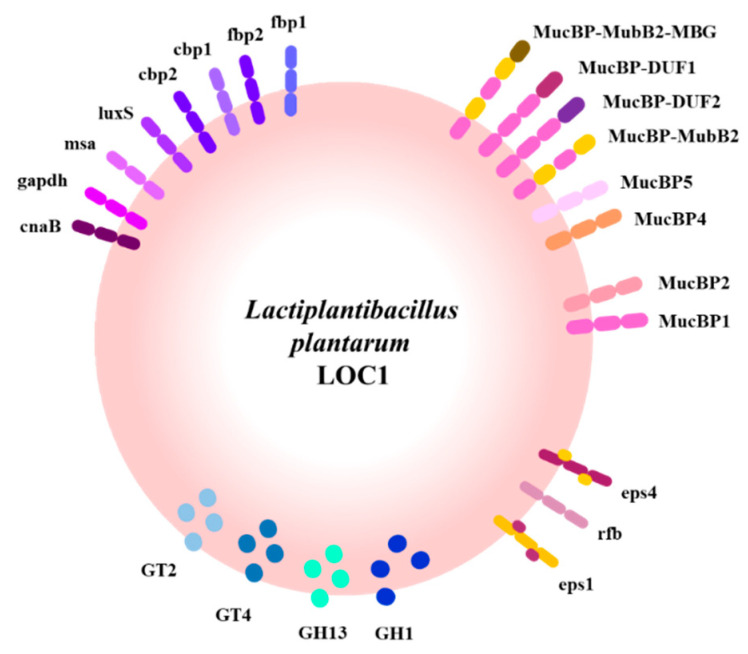
Schematic representation of glycosylhydrolases (GH), glycosyltransferases (GT), adhesion factors and exopolysaccharide (EPS) detected in the *Lactiplantibacillus plantarum* LOC1 genome. The genome of the LOC1 strain was studied and compared with the probiotic strains WCFS1 and CRL1506 as reference.

We performed here for the first time a genomic characterization of *L. plantarum* LOC1. Genomic comparative analysis was performed with the well-known immunomodulatory strains WCSF1 and CRL1506. We focused our analysis on the molecules of the bacterial surface that could be responsible for interacting with the host’s immunological receptors including surface proteins, adhesion factors, EPS and LTA. The bioinformatic study revealed that *L. plantarum* LOC1 possesses several factors that have been proposed to be important for the interaction of this species of bacteria with the host (Figure 13).

Some studies have shown that EPS produced by *L. plantarum* strains are involved in their immunomodulatory properties. The EPS from *L. plantarum* NCU116 was shown to reduce intestinal inflammation through the STAT3 signaling pathway [61], while the EPS from *L. plantarum* L-14 reduced the expression of cyclooxygenase-2, inducible nitric oxide synthase as well as *IL-6*, *TNF-α*, and *IL-1β* in LPS-challenged RAW macrophages [9]. Thus, we analyzed the EPS genes cluster in the LOC1 strain. There is high diversity among EPS clusters identified in *L. plantarum* strains. Five different EPS clusters were described in this species of lactobacilli [62]. In the model strain WSCF1, four major EPS-synthetic gene clusters were identified and designated as cps1, cps2, cps3, and cps4 [34]. Two separated regions of the *L. plantarum* WCFS1 genome contain those clusters: while cps1, cps2, and cps3 are located in tandem in one region, the cps4 cluster is located separated [34]. Of note, the cps1 gene cluster controlled the molecular mass and the rhamnose composition of EPS [62], while *cps4A-J* genes in the cps4 cluster regulate the overall EPS yield. In fact, the deletion of these genes resulted in a reduction of less than half fold EPS production compared to wild-type bacteria [34]. Of note, both clusters of EPS were almost complete in the genome of *L. plantarum* LOC1, while cps3 was not observed and cps2 partially detected. Similarly, it has been shown that not all *L. plantarum* strains had the four clusters of EPS genes in their genomes [28,63]. Strains ATCC14197 and ST-III have only the csp3 and csp4 clusters, JMD1 only possess the cps4 cluster [63], while the immunomodulatory strain CRL1506 carries cps2, cps3 and cps4 clusters [28]. This variability between the strains could impact in their interaction with the host and particularly with the cells of the immune system such as macrophages. Considering that the LOC1 strain presented differences in EPS clusters in relation to the immunomodulatory strain CRL1506 but modulated in a similar way the response of RAW macrophages, it is possible to speculate that EPS would not be involved in the immunomodulatory activity of *L. plantarum* LOC1.

We also investigated genes coding for proteins involved in the adhesion of *L. plantarum*, which are thought to be important for the strains to fulfill their probiotic functions. In the probiotic WCFS1 strain, several genes with adhesion function were observed, including mucin-, collagen-, chitin-, and fibronectin-binding proteins [29], as well as agglutination factors [64]. In a previous work, when adhesion factors were evaluated in different *L. plantarum* strains possessing differential immunomodulatory properties, we found a great variability between strains [28]. Similarly, we described here that *L. plantarum* LOC1 has its own characteristic set of adhesion factors (Figure 13). Assessing whether any of these factors are essential for its immunomodulatory activity is an interesting topic for future research.

In addition, genes involved in the biosynthesis of TA, LTA and lipoproteins were also analyzed in the genome of *L. plantarum* LOC1. Those molecules have been associated with the immunomodulatory properties of *L. plantarum* strains such as J26 and WCSF1 [36,65]. Thus, the lipoproteins-related genes (*lgt*, lp_0856, lp_0925 and lp_1181), the TA-related genes (*gtcA1*, *gtcA2*, *gtcA3*, *dltX*, *tagD1*, *tarI*, *tarJ*, *tarK* and *tarL*) and the LTA-related gene *dltD* of *L. plantarum* LOC1 were searched by in silico analysis. No differences were found between the LOC1 strain and *L. plantarum* WSCF1 and CRL1506, indicating that these molecules would be similar in the three strains and, therefore, are molecules that could be involved in the immunomodulatory capacity of *L. plantarum* LOC1.

When the genes belonging to metabolic pathways of the three strains (LOC1, WSCF1 and CRL1506) were compared, no significant differences were detected except for genes involved in carbohydrate metabolism, particularly in the presence/absence of GH and GT. These findings are in line with previous studies that reported differences in genes involved in sugar transport and catabolism among *L. plantarum* strains [66]. These different metabolic capacities condition the fermentation and growth characteristics of each strain [67] and collaborate in determining the types of habitats in which the bacteria will be able to subsist as well as their potential biotechnological applications. It is likely that these differences are noticeable in this work since the genomic studies concentrated on *L. plantarum* strains that come from different origins: fresh tea leaves, goat milk and human saliva. An interesting point for future research would be to carry out comparative genomic studies of *L. plantarum* LOC1 with strains isolated from plant sources and from fermented tea or tea fresh leaves. The sequencing of the LOC1 strain genome carried out in this work makes it possible to perform those studies as well as others that are necessary to advance in the potential biotechnological applications of this strain.

## 5. Conclusions

In the current study, we demonstrated that *L. plantarum* LOC1 isolated from fresh tea leaves is an interesting probiotic strain candidate with immunomodulatory activity. In vitro transcriptomic studies demonstrated that the LOC1 strain differentially modulates the expression of immune factors in LPS-challenged macrophages suggesting that this candidate probiotic has the capacity to improve the beneficial functions of these immune cells. In addition, we sequenced the complete genome of *L. plantarum* LOC1 and genomic studies showed that the strain possess several factors described for other probiotic strains that may be involved in its interaction with macrophages. The results of this work can contribute to the development of immune-related functional foods containing *L. plantarum* LOC1. Further investigation into the in vivo beneficial immunomodulatory effects of the LOC1 strain are ongoing.

## Figures and Tables

**Table 1:** General genomic characteristics of the *L. plantarum* LOC1. The genome of the LOC1 strain was studied and compared with the probiotic strains WCFS1 and CRL1506 as reference.

*Lactoplantibacillus plantarum*	LOC1	CRL1506	WCFS1
Host	*Camellia sinensis*	*Capra aegagrus hircus*	*Homo sapiens*
Origen	Fresh tea leaves	Milk	Saliva
Genome size (pb)	3,138,505	3,228,096	3,348,624
G+C content (%)	44.7	44.5	44.4
Genes	3078	3051	3154
Coding sequences (total)	2907	2967	3062
Protein coding sequences	2834	2918	3015
ARNr (5s, 16s, 23s)	3 (1, 1, 1)	13 (6, 4, 3)	16 (6, 5, 5)
ARNt	57	67	72
Access number	BOUN00000000	LNCP00000000	AL935263.2

## Data Availability

Data are contained within this article.

## Supplementary Materials

The following supporting information can be downloaded at: https://www.mdpi.com/article/10.3390/foods11203257/s1, Figure S1: Effect of *Lactiplantibacillus plantarum* strains isolated from fresh tea leaves in murine RAW macrophages stimulated with the Toll-like receptor 4 (TLR4) agonist lipopolysaccharide (LPS). Macrophages were treated with *L. plantarum* LOC1 or LOC3 strains and then challenged with LPS. The expression of genes was determined by microarray analysis 3 h after LPS stimulation. Non-lactobacilli-treated macrophages stimulated with LPS were used as control. Heat-map analysis and fold expression changes of surface markers (A) and interferons (IFNs) and IFNs-related genes (B). Asterisks indicate significant differences between the indicated groups and LPS-challenged control macrophages, (*) *p* < 0.05. Figure S2: Study of the number of genes in the different functional categories associated with “genetic information processing”, “environmental information processing” and “cellular process” pathways of the genome of *Lactiplantibacillus plantarum* LOC1. The genome of the LOC1 strain was studied and compared with the probiotic strains WCFS1 and CRL1506 as reference. Functional characterization of genes was performed with the BlastKOALA tool according to the KEGG database. Figure S3: Analysis of the presence/absence of genes involved in the biosynthesis of lipoproteins, teichoic acids and lipoteichoic acids in *Lactiplantibacillus plantarum* LOC1. The genome of the LOC1 strain was studied and compared with the probiotic strains WCFS1 and CRL1506 as reference. Table S1: Nucleotide sequences of the specific primers used for real-time PCR.
