# Supplementary material for: Lactiplantibacillus plantarum LOC1 Isolated from Fresh Tea Leaves Modulates Macrophage Response to TLR4 Activation
Source: Foods. 2022 Oct 18;11(20):3257. doi: 10.3390/foods11203257 (PMC9602255; doi:10.3390/foods11203257)
Supplement: Supplementary file 1 [file foods-11-03257-s001.zip › Figure S1.pptx]

## Slide 1
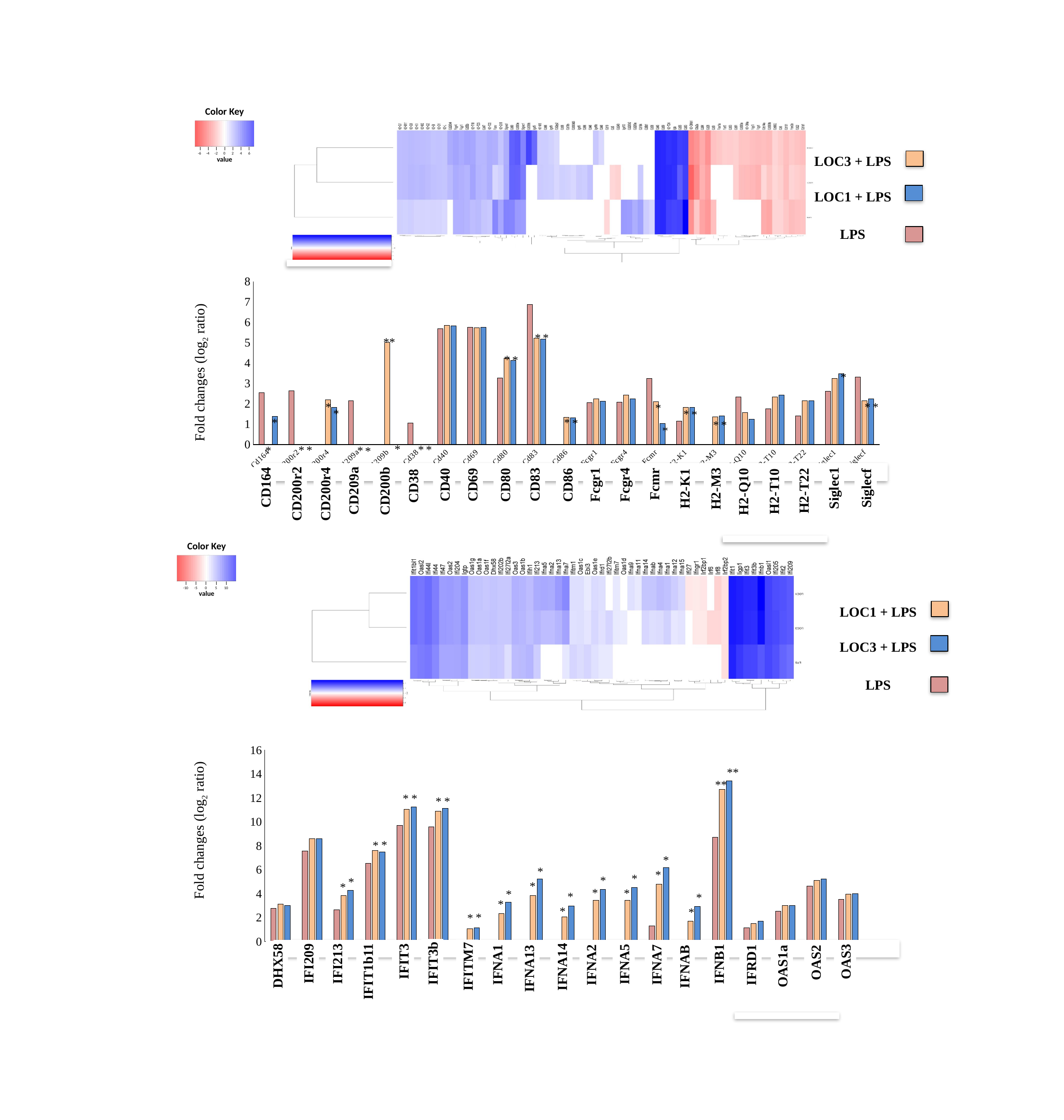

Color Key
| | | | | | | | |
| --- | --- | --- | --- | --- | --- | --- | --- |
| -6 | -4 | -2 | 0 | 2 | 4 | 6 |
| --- | --- | --- | --- | --- | --- | --- |
LOC3 + LPS
value
LOC1 + LPS
LPS
### Chart
| Category | LPS | LOC3 | LOC1 |
|---|---|---|---|
| Cd164 | 2.5442443 | 0.0 | 1.3742685 |
| Cd200r2 | 2.64253 | 0.0 | 0.0 |
| Cd200r4 | 0.0 | 2.1913671 | 1.8228402 |
| Cd209a | 2.15722079999999 | 0.0 | 0.0 |
| Cd209b | 0.0 | 5.020059 | 0.0 |
| Cd38 | 1.0642104 | 0.0 | 0.0 |
| Cd40 | 5.675788 | 5.8570557 | 5.832567 |
| Cd69 | 5.760333 | 5.73831459999999 | 5.7650194 |
| Cd80 | 3.2594838 | 4.2383327 | 4.128257 |
| Cd83 | 6.8721166 | 5.229248 | 5.163336 |
| Cd86 | 0.0 | 1.33596989999999 | 1.3114986 |
| Fcgr1 | 2.0555506 | 2.2353725 | 2.12453079999999 |
| Fcgr4 | 2.0671358 | 2.4309006 | 2.2470255 |
| Fcmr | 3.2295604 | 2.101883 | 1.0358953 |
| H2-K1 | 1.1500225 | 1.8150921 | 1.8181915 |
| H2-M3 | 0.0 | 1.3439808 | 1.3982353 |
| H2-Q10 | 2.32370379999999 | 1.5634909 | 1.236701 |
| H2-T10 | 1.7618685 | 2.3427715 | 2.422061 |
| H2-T22 | 1.4074316 | 2.1402245 | 2.1445646 |
| Siglec1 | 2.61064 | 3.2440095 | 3.481112 |
| Siglecf | 3.3058982 | 2.1489944 | 2.232524 |*
*
**
*
*
Fold changes (log2 ratio)
*
*
*
*
*
*
*
*
*
*
*
*
*
*
*
*
*
*
*
*
*
*
CD164
Fcgr4
Fcgr1
CD83
CD86
CD80
CD38
CD40
CD209a
Siglec1
H2-M3
CD200r2
CD200b
Fcmr
H2-Q10
CD69
H2-T22
Siglecf
H2-T10
H2-K1
CD200r4
Color Key
| | | | | | |
| --- | --- | --- | --- | --- | --- |
| -10 | -5 | 0 | 5 | 10 |
| --- | --- | --- | --- | --- |
value
LOC1 + LPS
LOC3 + LPS
LPS
### Chart
| Category | LPS | LOC3 | LOC1 |
|---|---|---|---|
| Dhx58 | 2.78040029999999 | 3.1234045 | 3.0119672 |
| Ifi209 | 7.5502815 | 8.578526 | 8.60254 |
| Ifi213 | 2.6446285 | 3.837037 | 4.2882185 |
| Ifit1bl1 | 6.528382 | 7.586512 | 7.4772906 |
| Ifit3 | 9.703583 | 11.032979 | 11.236982 |
| Ifit3b | 9.578332 | 10.894328 | 11.135114 |
| Ifitm7 | 0.0 | 1.0735435 | 1.1509762 |
| Ifna1 | 0.0 | 2.3534355 | 3.272359 |
| Ifna13 | 0.0 | 3.84216169999999 | 5.231472 |
| Ifna14 | 0.0 | 2.0517983 | 2.99056429999999 |
| Ifna2 | 0.0 | 3.4531507 | 4.3529468 |
| Ifna5 | 0.0 | 3.435894 | 4.530258 |
| Ifna7 | 1.29612969999999 | 4.779618 | 6.183199 |
| Ifnab | 0.0 | 1.6919346 | 2.94820449999999 |
| Ifnb1 | 8.698807 | 12.696144 | 13.4240339999999 |
| Ifrd1 | 1.1566896 | 1.500761 | 1.7092791 |
| Oas1a | 2.53062439999999 | 3.0074234 | 3.0251675 |
| Oas2 | 4.6216383 | 5.113331 | 5.2401586 |
| Oas3 | 3.5295286 | 3.949934 | 4.0004063 |**
**
*
*
*
*
Fold changes (log2 ratio)
*
*
*
*
*
*
*
*
*
*
*
*
*
*
*
*
*
*
*
*
IFIT3b
IFNA5
IFNA1
IFNA2
DHX58
IFNA14
IFITM7
OAS3
IFNB1
IFI209
IFIT3
IFNA7
IFNA13
IFRD1
OAS2
OAS1a
IFNAB
IFIT1b11
IFI213
