# Supplementary material for: Lactiplantibacillus plantarum LOC1 Isolated from Fresh Tea Leaves Modulates Macrophage Response to TLR4 Activation
Source: Foods. 2022 Oct 18;11(20):3257. doi: 10.3390/foods11203257 (PMC9602255; doi:10.3390/foods11203257)
Supplement: Supplementary file 1 [file foods-11-03257-s001.zip › Figure S2.pptx]

## Slide 1
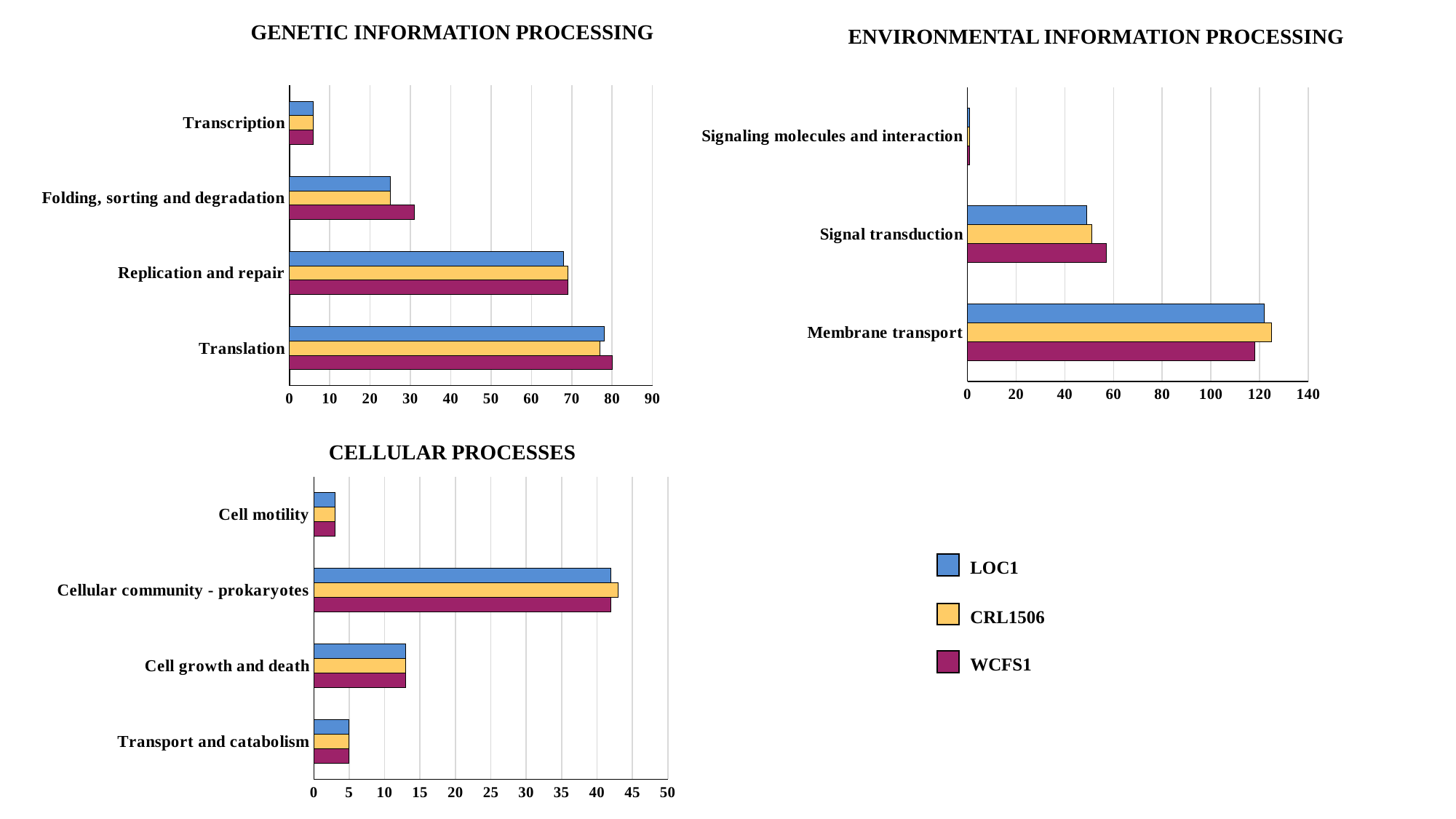

GENETIC INFORMATION PROCESSING
 ENVIRONMENTAL INFORMATION PROCESSING
### Chart
| Category | WCFS1 | CRL1506 | LOC1 |
|---|---|---|---|
| Translation | 80.0 | 77.0 | 78.0 |
| Replication and repair | 69.0 | 69.0 | 68.0 |
| Folding, sorting and degradation | 31.0 | 25.0 | 25.0 |
| Transcription | 6.0 | 6.0 | 6.0 |
### Chart
| Category | WCFS1 | CRL1506 | LOC1 |
|---|---|---|---|
| Membrane transport | 118.0 | 125.0 | 122.0 |
| Signal transduction | 57.0 | 51.0 | 49.0 |
| Signaling molecules and interaction | 1.0 | 1.0 | 1.0 |CELLULAR PROCESSES
### Chart
| Category | WCFS1 | CRL1506 | LOC1 |
|---|---|---|---|
| Transport and catabolism | 5.0 | 5.0 | 5.0 |
| Cell growth and death | 13.0 | 13.0 | 13.0 |
| Cellular community - prokaryotes | 42.0 | 43.0 | 42.0 |
| Cell motility | 3.0 | 3.0 | 3.0 |LOC1
CRL1506
WCFS1
