# Supplementary material for: Lactiplantibacillus plantarum LOC1 Isolated from Fresh Tea Leaves Modulates Macrophage Response to TLR4 Activation
Source: Foods. 2022 Oct 18;11(20):3257. doi: 10.3390/foods11203257 (PMC9602255; doi:10.3390/foods11203257)
Supplement: Supplementary file 1 [file foods-11-03257-s001.zip › Figure S3.pptx]

## Slide 1
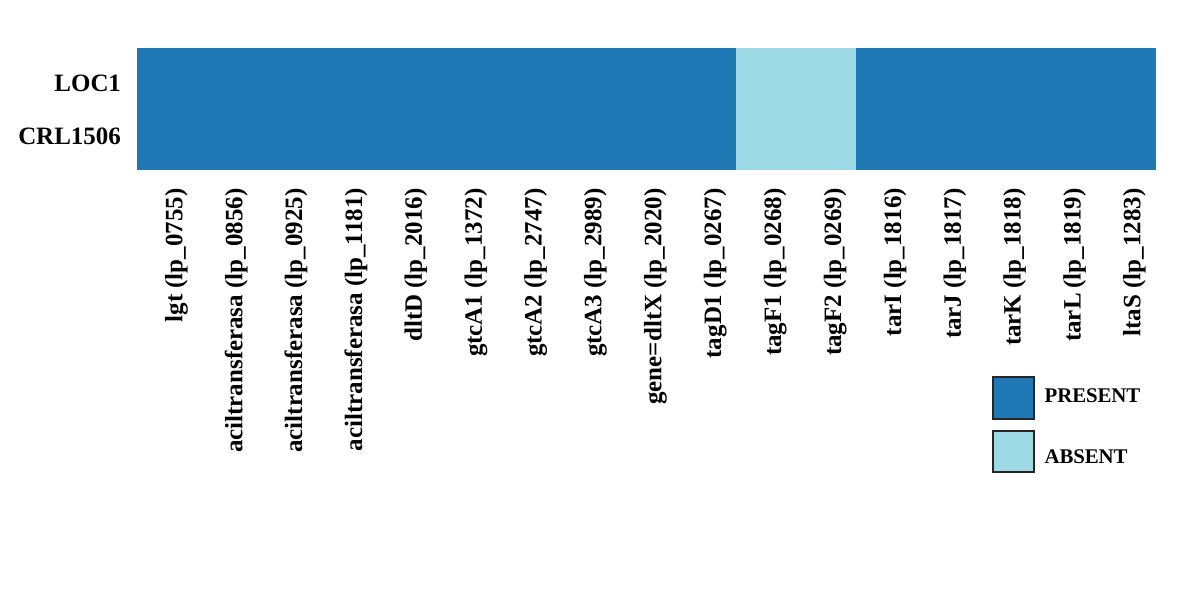

lgt (lp_0755)
aciltransferasa (lp_0856)
aciltransferasa (lp_0925)
aciltransferasa (lp_1181)
dltD (lp_2016)
gtcA1 (lp_1372)
gtcA2 (lp_2747)
gtcA3 (lp_2989)
gene=dltX (lp_2020)
tagD1 (lp_0267)
tagF1 (lp_0268)
tagF2 (lp_0269)
tarI (lp_1816)
tarJ (lp_1817)
tarK (lp_1818)
tarL (lp_1819)
ltaS (lp_1283)
| | | | | | | | | | | | | | | | | |
| --- | --- | --- | --- | --- | --- | --- | --- | --- | --- | --- | --- | --- | --- | --- | --- | --- |
| | | | | | | | | | | | | | | | | |
LOC1
CRL1506
PRESENT
ABSENT
